# Personal News Items

**Published:** 1915-09

**Authors:** 


### Personal News Items.

Dr. C. M. Grigsby, of Dallas, has returned home after taking a post-graduate course in Boston.

Dr. and Mrs. H. R. Gilliam, of Houston, have returned home from the Exposition.

Dr. M. M. Carrick, of Dallas, has declined the position of health commissioner of the Republic of Mexico, recently tendered him by the government.

Dr. and Mrs. Cassell, of Lampasas, are spending a few weeks in San Antonio.

Dr. Thomas Paisley Williams, President of the Texas Dental College, at Houston, has built him a magnificent home in Park Place, where he expects to move early in September.

Dr. and Mrs. T. W. Foster, of Marlin, are visiting in San Antonio.

Dr. and Mrs. C. E. Scull, of San Antonio, are in New York this month.

Dr. and Mrs. Roy Colston Miner, of Charlottesville, Va., are visiting Dr. and Mrs. Charles Venable in San Antonio.

Dr. S. L. Stalling has returned to his home in Pearsall after a several weeks' stay in Corpus Christi.

Dr. and Mrs. R. C. Fisher, of Belton, left recently for the San Francisco Exposition.

Dr. M. D. Baker and family, of Waco, are visiting in Albany.

Dr. and Mrs. R. L. Brown, of Dilley, are attending the California Expositions.

Dr. and Mrs. J. S. Turner, accompanied by Dr. and Mrs. A. L. Frew, of Dallas, left recently for a few weeks' camp at Medina dam and other camping places.

Dr. G. M. Callaway and family have returned to their home in El Paso after a short visit to Rockport and other points on the Gulf.

Dr. Ladd and family of Mesilla Park have returned home from the San Francisco Exposition.

Dr. and Mrs. C. T. Gray, of Austin, are spending the summer in Chicago, where Dr. Gray is taking work in the Chicago University.

Dr. and Mrs. W. R. Manning, of Austin, are spending the summer in California.

Dr. and Mrs. Carl Hartman, of Austin, are in Chicago, where Mrs. Hartman has undergone a serious operation, from wihch she is now recovering.

Dr. Henry C. Evans returned recently to his home, Milford, from Colorado Springs, Colo., where he has been spending the summer.

Dr. and Mrs. J. M. Gober, of Beaumont, have returned from a five weeks' trips to the Pacific coast.

Dr. Scarbrough, of Fort Worth, is spending two weeks in Abilene.

Dr. and Mrs. E. B. Keener, of Galveston, are spending the summer in California and the Pacific coast.

Dr. J. E. Robinson, of Temple, has returned from a visit North, where he sought health benefit.

Dr. J. B. Shelmire, of Dallas, is attending the clinics of the Harvard Post-Graduate School.

Dr. and Mrs. S. M. Hill, of Dallas, have returned from Galveston and other coast points.

Dr. Brown and wife, of Wallace, are visiting in Port Lavaca.

Dr. J. A. Collins, of Post City, is spending his vacation in San Francisco.

Dr. and Mrs. D. M. Foster, of Leonard, Texas, are visiting in El Paso.

Dr. R. H. Stewart, of Waco, is spending the summer in California and the coast country.

Dr. W. H. Lawrence, of Aransas Pass, is spending a few weeks in Bryan.

Dr. W. E. Watt, of the city hospital of Memphis, is spending his vacation in Austin with his parents, Dr. and Mrs. W. N. Watt.

Dr. Griff Ross, who recently graduated from the Medical Department at Baylor, will start practice soon in Mt. Vernon.

Dr. and Mrs. Albert Grant, of Austin, have returned home from the Exposition.

Dr. Paul Cook, of Uvalde, went to San Antonio recently and secured some additional hospital equipment for his operating room, which he has recently fitted up at his residence. He already has a well equipped operating room with modern operating table, sterilizing plant for instruments and dressing, and other fixtures and conveniences incident to modern hospital work.

He plans to offer the advantages of his improvements to other practitioners of Uvalde and other towns in that vicinity, and states that some of the San Antonio surgeons have agreed to go there and operate under the same terms and conditions as they would operate in San Antonio.

Dr. and Mrs. F. W. Hover, of Sealy, have returned from New York, where Dr. Hover has been taking a course at the New York Post-Graduate Hospital.

Dr. W. B. Carrell, of Dallas, has returned from a two months' trip to Boston.

Dr. R. S. Yancy, of Dallas, left recently for a month's stay in the North and East.

Dr. and Mrs. J. G. Guenther, of Hallettsville, returned recently from San Francisco.

Dr. W. E. Tatum, of Beaumont, has returned from Chicago and Rochester, Minn., where he has been taking a post-graduate course, and will be associated with Dr. J. W. Garth.

Dr. F. J. Roemer and wife, of Port Lavaca, have left for Memphis and New York, where Dr. Roemer will take a post-graduate course in medicine.

Dr. and Mrs. James Blair, of Dallas, are spending the summer in Los Angeles and San Francisco.

Dr. and Mrs. W. T. Shell, of Corsicana, are in New York City, where Dr. Shell is taking a post-graduate course in medicine.

Dr. A. F. Beverly has returned to his home in Austin after a visit to the expositions in California.

Dr. and Mrs. M. T. Hendrics, of Lockhart, have returned from the Panama Exposition and a tour of the West.

Dr. Starley, of Galveston, left recently to attend clinics at Rochester, Minn., and Chicago.

Dr. Joseph Trice has returned to his home in Terrell after a several days' visit to Oklahoma.

Friends will be glad to learn that Dr. J. A. Yeager, of Rose Hill, who has been undergoing treatment at Fort Worth, is very much improved and will be able to return to his home soon.

Dr. Rosalie McAdams, of Bryan, formerly connected with Dr. Talbot's Sanitarium staff in that city, has accepted an offer to become associated with the surgical staff of the Taylor Sanitarium.

Dr. J. Hal Gambrell, of Dallas, has been appointed surgeon for the Cotton Belt Railway Company.

Dr. John S. Turner has been appointed a member of the Dallas Board of Health by Mayor Henry D. Lindsley. Dr. Turner succeeds Dr. J. O. McReynolds, who recently resigned.

At the monthly meeting of the Board of Managers of the Confederate Home, Dr. J. M. G. Gill, of Cameron, was appointed physician of the Home in place of Dr. T. F. Moore, who resigned to attend college at New York.

Dr. Belle Eskridge and Dr. Ray K. Daily, of Houston, are spending their vacation in California.

Dr. B. L. Lockett, medical missionary in Oyo-Logo, West Africa, has arrived in Dallas, and for several weeks will be the guests of Dr. and Mrs. B. E. Compre.

Dr. E. W. Loomis, Assistant City Health Officer of Dallas, has recently delivered a series of unusually interesting lectures on "Contagious Diseases" to the Mothers' Clubs of Dallas.

Dr. Burdett L. Arms, Professor of Preventive Medicine of the University of Texas, and Dean of the Medical Department at Galveston, delivered a lecture on "Bacteria and Pure Milk" in the roof garden patio of the Gunter Hotel, Thursday, August 5th. At this meeting, Dr. E. A. Golaz, State Chemist, connected with the Pure Food Department, also delivered a lecture on handling the milk in the home.

Dr. J. G. Webb, formerly Professor of Physiology in the Medical Department of the Texas Christian University of Fort Worth, has taken up the practice of medicine at Mercedes.

Among the first persons in Dallas to receive intelligence of the storm at Galveston was Dr. I. F. Orton, President of the Interstate Chemical Company of Galveston, who was in Dallas on business. In order that he might reach home in time to remove his family from the island if such a step should be necessary, Dr. Orton ordered a special train on the Santa Fe Railroad.

